# Circular RNA 0001823 aggravates the growth and metastasis of the cervical cancer cells through modulating the microRNA-613/RAB8A axis

**DOI:** 10.1080/21655979.2022.2063665

**Published:** 2022-04-17

**Authors:** Hong Ji, Naijun Hu

**Affiliations:** a Department of Obstetrics and Gynecology, The First Affiliated Hospital of Jinzhou Medical University, Jinzhou, Liaoning, China; b Department of General Surgery, The First Affiliated Hospital of Jinzhou Medical University, Jinzhou, Liaoning, China

**Keywords:** Cervical cancer, miR-613, RAB8A, proliferation, metastasis

## Abstract

Cervical cancer (CC) is a gynecological cancer, which has become the second malignant tumor with mortality in developing countries. The purpose of current study was to explore the influence of Circular RNA 0001823 (circ_0001823) in the CC development. Thirty CC tissues and paracancerous tissues were obtained, and Hela and CaSki CC cells were purchased for this study. The cell growth was analyzed by CCK-8 and colony formation assays. The cell metastasis was determined with Transwell assay. The circ_0001823, miR-613, and RAB8A expression were analyzed with qRT-PCR analysis. The specific mechanisms of circRNA_0001823 were analyzed by Dual luciferase reporter and RNA pull-down assays. The circ_0001823 and RAB8A expressions were increased, and miR-613 were decreased in the CC cells and tissues. Knockdown of circ_0001823 inhibited the malignant behavior of the CC cells, which was antagonized by miR-613 inhibitor. Over-expressed RAB8A reversed the miR-613 effects in the CC cells. Knockdown of circ_0001823 inhibited the malignant behaviors of the CC cells via regulating the miR-613/RAB8A axis.

## Graphical abstract

**Figure:**
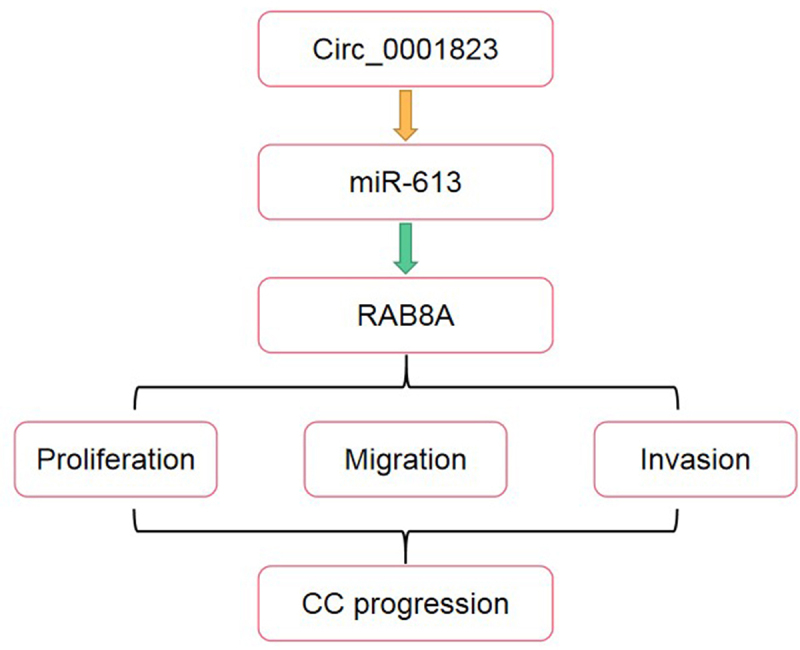

## Introduction

Cervical cancer (CC) is a common gynecological cancer, which has become the second malignant tumor with mortality in developing countries [1]. Previous study confirmed the persistent infection of high-risk human papillomavirus (HR-HPV) is the main cause for the occurrence and development of CC [2]. In recent years, with the in-depth study of HPV vaccine, the primary prevention of CC has achieved remarkable development, and its incidence rate and mortality rate has dropped significantly [3]. However, HPV infection cannot accurately predict the occurrence of CC. It not only brings a heavy burden to the diagnosis and prevention of CC, but also indicates that there are other important factors such as genetic differences among individuals in the development of CC [4,5]. Hence, it is very important to investigate the specific mechanism of CC progression and explore a novel diagnostic marker for CC treatment.

Only about 2% of the nucleic acid sequences of the human genome were converted into proteins. Most genes are transcribed into noncoding RNA (ncRNA) [6]. Recently, circular RNA (circRNA) is a new research hotspot and attracted extensive attention of scholars at home and abroad [7]. In 1976, Sanger and kolakofsky first demonstrated the existence of circRNA in plant viroid and Sendai virus [8,9]. Subsequently, HUS clearly observed the circular structure of circRNA by electron microscope in 1979 [10]. At present, based on the rapid development of sequencing technology and gene chips, many new advances have been made in the research of circRNA. Li et al. [11] first found hsa_circ_002059 was down-regulated in gastric cancer and closely related to the distal metastasis, lymph node metastasis, patient gender and age. Chen et al. [12] found hsa_circ_0000190 was a marker of gastric cancer, which exhibited high sensitivity and specificity. Wang et al. [13] demonstrated that hsa_circ_001988 was dramatically down-regulated in colorectal cancer, and it participated in the regulation of differentiation level of colon cancer cells and neurophilic invasion. It can be seen that specific circRNA plays are involved in tumorigenesis and development, and function as molecular markers for tumor diagnosis and prognosis assessment.

RAB8 is a member of RAB protein family. It is a multifunctional GTPase, which can be coupled with a variety of effectors to act on different cellular pathways [14]. RAB8 is an important protein in the process of protein transportation from Golgi apparatus to plasma membrane. RAB8 protein includes RAB8A and RAB8B, of which RAB8A is the main type [15]. It has been found that RAB8A participates in the regulation of Golgi cell membrane post transport, reverse transcriptase mediated transport, vesicle transport, and exocytosis [16]. However, the role of RAB8A in the CC development remains unclear.

Therefore, in the present study, we confirmed that circ_0001823 was over-expressed in CC via bioinformatic analysis. The purpose of this study was to investigate the specific mechanism of circ_0001823 in CC progression, and provided a novel therapeutic treatment for CC. We hypothesized that circ_0001823 regulated the CC progressions via targeting the miR-613/RAB8A axis.

## Material and method

### Patients

Thirty CC tissues and paracancerous tissues were obtained from the patients in the First Affiliated Hospital of Jinzhou Medical University. The clinicopathologic characteristics of study subjects are shown in Table 1. The samples are stored at −80°C for the further experiments. All participants agreed to this experiment and signed the consent form. Besides, this research was approved by the Committee of the First Affiliated Hospital of Jinzhou Medical University Hospital.

**Table 1.** Clinicopathologic characteristics of study subjects

Clinicopathologic characteristics	*n*	Low	High	*p*-Value
Age (years)				0.6052
<55	13	6	7	
≥55	18	10	8	
Sex				0.8528
Male	15	8	7	
Female	16	8	8	
AJCC stage				0.0451*
I+ II	14	10	4	
III+IV	17	6	11	
T stage				0.0484*
T1 + T2	13	4	9	
T3 + T4	18	12	6	
Tumor size				0.5518
<40 mm	12	7	5	
≥40 mm	19	9	10	
Lymph node metastasis				0.0126*
Yes	11	9	2	
NO	20	7	13	

## Cell culture and transfection

The Human normal cervical epithelial cells (HNCEC) and human cervical cancer cell lines (SiHa, Hela, CaSki, C33A, and SW756) were provided by CHI Scientific company (MA, USA). All cells were added with Dulbecco’s Modified Eagle’s Medium medium supplemented with 10% fetal bovine serum (Invitrogen) and 2.5 mM L-glutamine (Sigma-Aldrich, MO, USA), then cultured at 37°C with 5% CO2.

ShRNA circ_0001823 (sh-circ_0001823), miR-613 inhibitor, miR-613 mimic, sh-RAB8A, and oe-RAB8A and sh-nc, inhbitor or mimic nc and vector were provided by Shanghai GenePharma Co., Ltd. (Shanghai, China) [17]. All these plasmids were transfected into the HeLa and CaSki cells with Lipofectamine 2000 (Thermo Fisher Scientific, MA, USA).

## Quantitative real-time PCR (qRT-PCR)

The RNA was obtained by using TRIzol reagent (Beyotime, Shanghai, China). Next, we measured the purity and concentration of the extracted RNA using nanodrop1000. Then, a reverse transcription kit (Takara, Tokey, Japan) was purchased to obtain cDNA. After that, the qRT-PCR was performed with a SYBR® Premix Ex Taq^TM^ kit (Takara) with the program of 95°C, 10 min; 95°C, 15 s, 35 cycles; 60°C, 20 s; 72°C, 45 s. All samples were tested in triplicate. GAPDH was selected as housekeeping gene. The relative mRNA expression was quantified with 2^−ΔΔCt^ method [18].

In addition, 2 mg/mL Actinomycin D (Sigma, NJ, USA) and 40 U RNase R (Sigma) were selected to detect the circ_0001823 stability with qRT-PCR analysis.

## Determination of cell viability

The cells were incubated in a 96-well plate (2 × 10^3^ cells/well) for 24 hours. Next, the cells were incubated with 10 μl CCK-8 solution (GLPBIO, USA) in the dark for another 2 hours (37°C) [19]. Finally, the absorbance was determined at 450 nm wavelength by a Model 680 microplate reader (Bio-Rad).

## Colony formation assay

The cells were cultured in 6-well plates for 2 weeks without medium change. After that, the cells were fixed by 4% paraformaldehyde, and then stained by .4% crystal violet [20]. Finally, the microscope was used to analyze the number of cloned cells

## Determination of migration and invasion abilities

First, 48-well plates were put into the generated upper and lower chambers of the cell culture Transwell inserts. Additionally, cells were placed into the upper chamber with a Matrigel-coated membrane to detect the migrated cells, or not coated with Matrigel to detect invaded cells, while the lower chambers were added with culture media supplement with serum. After 24 hours, the number of migrated and invaded cells was calculated [21].

## Dual luciferase reporter assay

The circ_0001823 3’-UTR wild-type (wt) and mutant-type (mut), RAB8A 3’-UTR wt, and mut were purchased and cloned into fluorescent vector psiCHECK2 to analyze whether miR-613 directly targets circ_0001823 or RAB8A 3’-UTR. miR-613 mimic, miR-613 mimic nc and Renilla luciferase plasmid (Promega, Madison, USA) were co-transfected into the cells. Luciferase activity was detected consecutively by a Dual-Luciferase Reporter Assay kit [22].

## RNA pull-down assay

The cell was lysed and incubated with biotinylated miR-613 (biotin-miR-613) or biotin-nc. The biotin-miR-613 concentration was 20 nM. After 2 days, the cell lysates were obtained and added with M-280 Streptavidin magnetic beads for 3 hours. Next, after washing three times, TRIzol was used to purify the RNA. After qRT-PCR was conducted to quantify the circ_0001823 and RAB8A expressions [23].

## Statistics analysis

The data in this study were analyzed using SPSS22.0 and expressed as mean ± SD. The student T-test was used for analyzing the difference between the two groups, while ANOVA followed by with Duncan’s post-hoc test was selected for multiple groups. In addition, bioinformatics tools were performed to obtained volcano map of the differentially expressed circRNAs from the GEO database. P < 0.05 was meant a significant difference.

## Results

This study demonstrated that circ_0001823 was up-regulated in CC via bioinformatic analysis, which was further confirmed to be over-expressed in CC tissues as well as cells. Knockdown of circ_0001823 inhibited the growth and matastasis of the CC cells through modulating the miR-613/RAB8A axis.

## Circ_0001823 was up-regulated in CC tissues as well as cells

**Figure 1.**
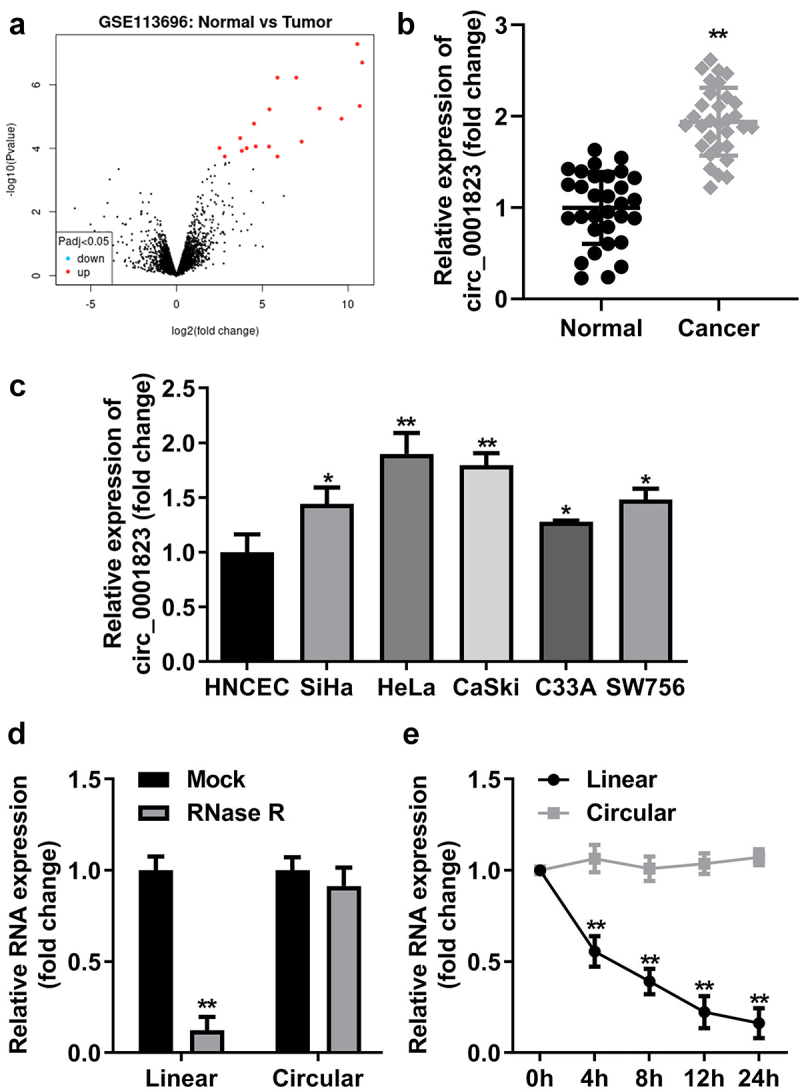
Circ_0001823 was over-expressed in CC tissues as well as cells.A Volcano map showed significant differentially expressed circRNAs via log2-fold change and log10 p-values. The circ_0001823 expression in the CC patients (b) and CC cells (c) were measured by qRT-PCR asssay. D-E Verification of circ_0001823 stability. *P < 0.05. **P < 0.01.

First, through volcano map, we found the circ_0001823 expression was the highest in the tumor compared with the normal tissue (Figure 1a). In CC patients, circ_0001823 expression was dramatically up-regulated (Figure 1b), which was also observed in CC cell lines. Circ_0001823 expression was highest in HeLa and CaSki cells. Therefore, we selected HeLa and CaSki cells for the further experiments (Figure 1c). Then, the results of RNase R treatment showed the expression level of lnc_0001823 was substantially decreased compared with circ_0001823 (Figure 1d). Circ_0001823 exhibited more stable expression compared with linear group (Figure 1e).

## Circ_0001823-silenced inhibited the malignant behavior of the CC cells

**Figure 2.**
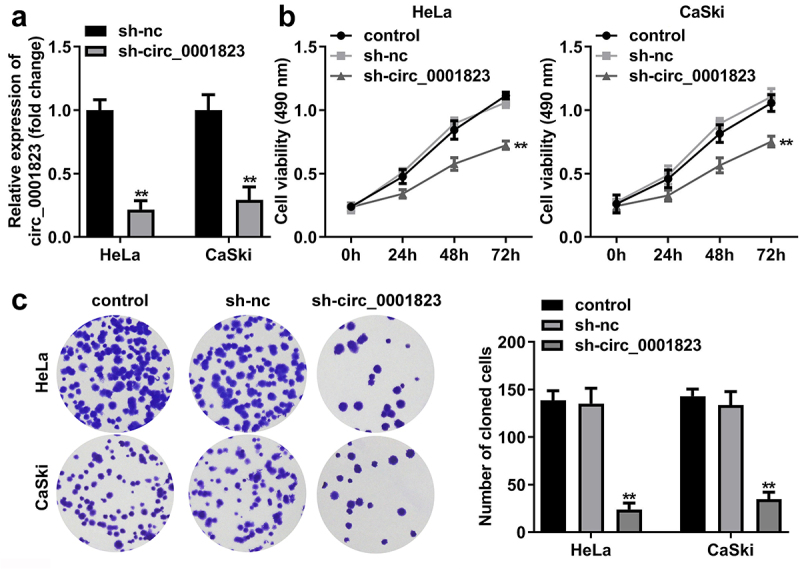
Circ_0001823 knockdown inhibited the proliferation of the CC cells. A Validation of sh-circ_0001823 transfection efficiency. B-C After sh-circ_0001823 transfection, CCK-8 and colony formation assays were performed to measure the cell viability and cloned cells numbers. **P < 0.01.

After sh-circ_0001823 transfection, the circ_0001823 expression was dramatically down-regulated in the HeLa and CaSki cells (Figure 2a). The cell viability and the number of cloned cells of the HeLa and CaSki cells were significantly down-regulated after sh-circ_0001823 transfection (Figure 2(b,c)).

**Figure 3.**
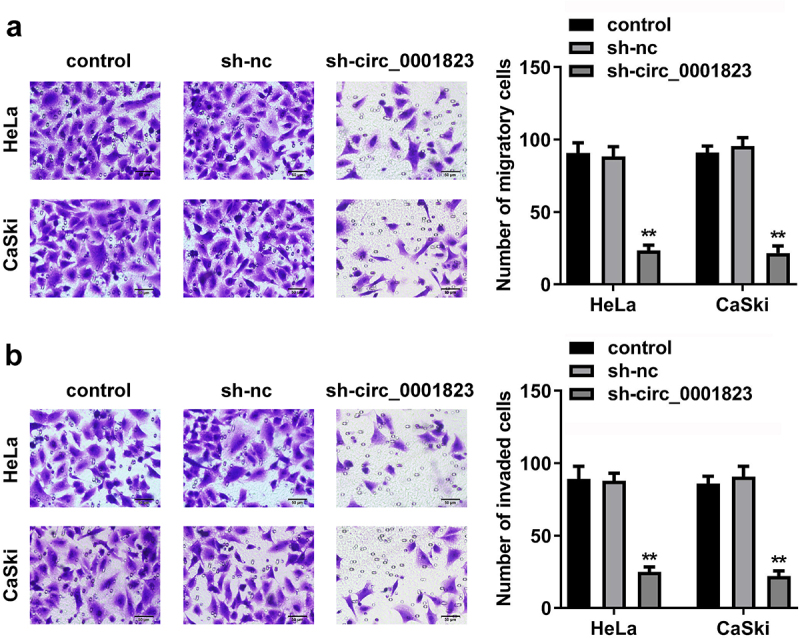
Circ_0001823-silenced inhibited the malignant behavior of the CC cells. A-B After sh-circ_0001823 transfection, transwell assay was conducted to analyze the migration and invasion of the CC cells . **P < 0.01.

As shown in Figure 3(a,b), the migrated and invaded cells in the HeLa and CaSki cells were dramatically down-regulated after sh-circ_0001823 transfection.

## Circ_0001823-overexpressed promoted the malignant behavior of the CC cells

**Figure 4.**
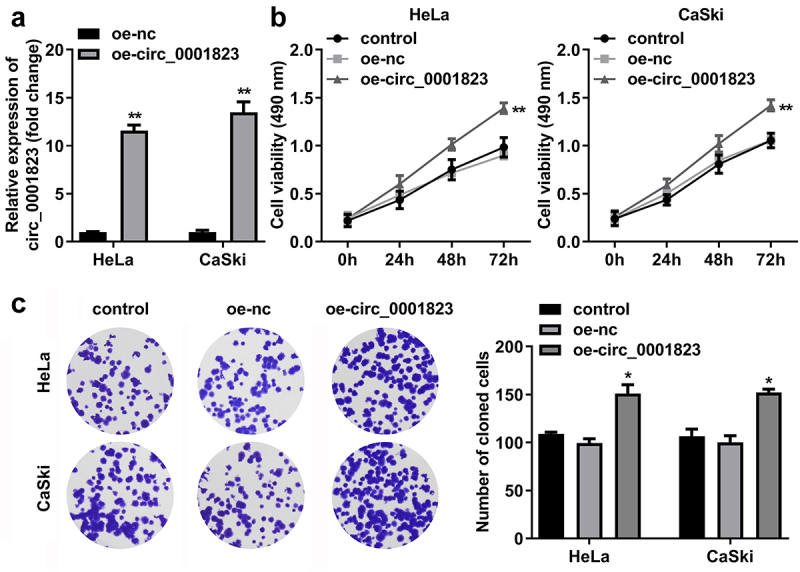
Circ_0001823 overexpression promoted the proliferation of the CC cells. A Validation of oe-circ_0001823 transfection efficiency. B-C After oe-circ_0001823 transfection, CCK-8 and colony formation assays were performed to measure the cell viability and cloned cells numbers. *P < 0.05. **P < 0.01.

After oe-circ_0001823 transfection, the circ_0001823 expression was dramatically up-regulated in the HeLa and CaSki cells (Figure 4(a)). The cell viability and the number of cloned cells of the HeLa and CaSki cells were significantly up-regulated after sh-circ_0001823 transfection (Figure 4(b,c)).

**Figure 5.**
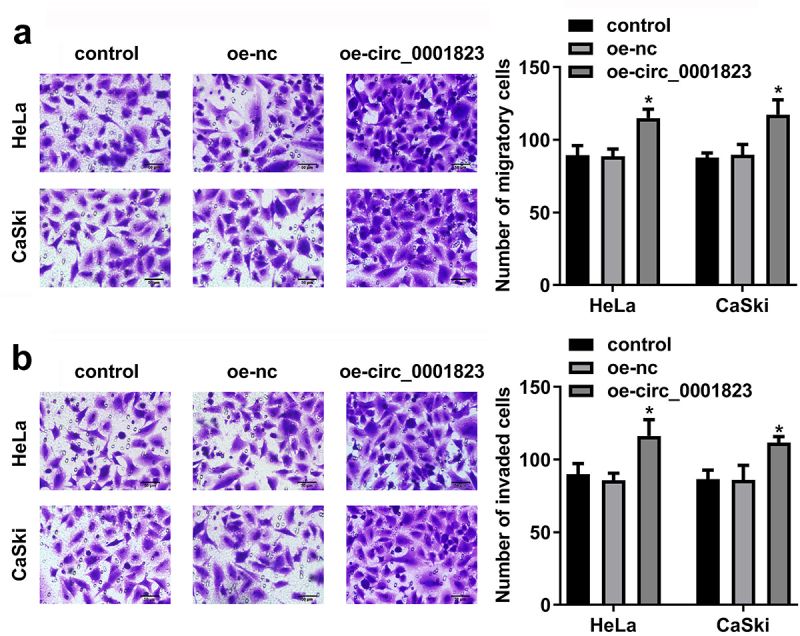
Circ_0001823-overexpressed promoted the malignant behavior of the CC cells. A-B After oe-circ_0001823 transfection, transwell assay was conducted to analyze the migration and invasion of the CC cells . *P < 0.05.

As shown in Figure 5(a,b), the migrated and invaded cells in the HeLa and CaSki cells were dramatically up-regulated after oe-circ_0001823 transfection.

## Circ_0001823 acted as a miR-613 sponge in CC cells

**Figure 6.**
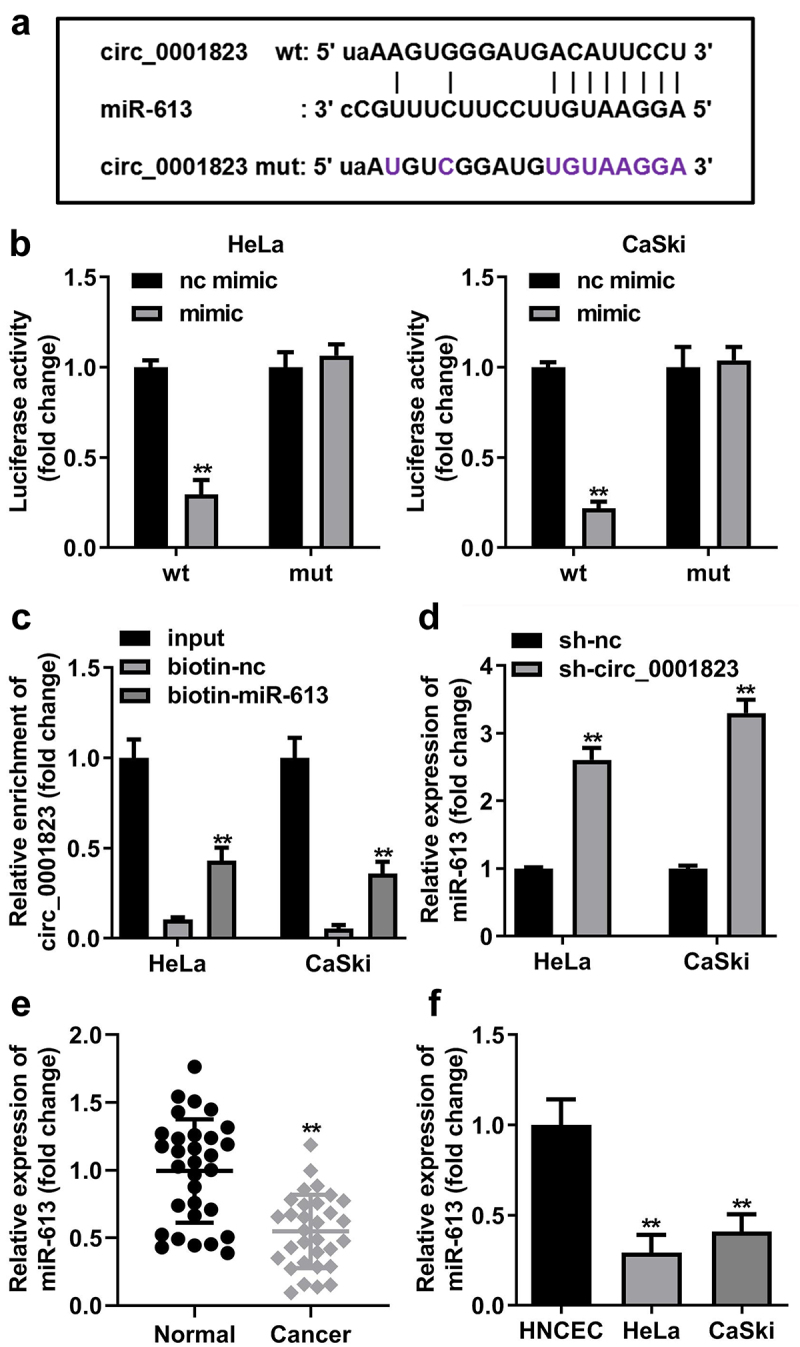
Circ_0001823 acted as a miR-613 sponge in CC cells. A The predicted circ_0001823 binding site in the miR-613 3’-UTR. B-C Double Luciferase Report and RNA pull-down assays were carried out to confirmed circ_0001823 could bind to miR-613. D The miR-613 expression in the CC cells was measured with qRT-PCR assay after sh-circ_0001823 transfection. E-F The miR-613 expression in the CC tissues as well as cells was detected with qRT-PCR assay. **P < 0.01.

The binding site between circ_0001823 and miR-613 was obtained with online database Starbase (Figure 6(a)). In addition, the luciferase activity was dramatically attenuated in the HeLa and CaSki cells incubated with circ_0001823 3’ UTR wt (Figure 6(b)). In RNA-pull down assay, miR-613 enriched evidently higher level of circ_0001823 in the CC cells (Figure 6(c)). Besides, after sh-circ_0001823 transfection, the miR-613 expression was dramatically increased in the CC cells (Figure 6(d)). Furthermore, we found that miR-613 expression was dramatically decreased both in the CC tissues and cells (Figure 6e and 6f).

## miR-613 inhibitor treatment reversed the role of sh-circ_0001823 in the CC cells

**Figure 7.**
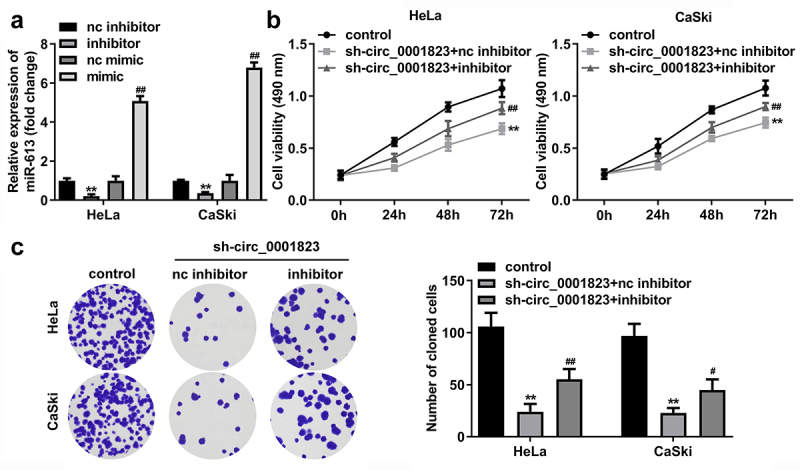
miR-613 inhibitor treatment reversed the effects of sh-circ_0001823 on the cell viability as well as cloned cells numbers in the CC cells.A Validation of miR-613 inhibitor or mimic transfection efficiency. B-C After sh-circ_0001823 and miR-613 inhibitor transfection, the cell viability and cloned cells numbers were measured by CCK-8 and colony formation assays. **P < 0.01 VS control group. #*P* < 0.05, ##*P* < 0.01 VS sh-circ_0001823+ miR-613 inhibitor nc group.

**Figure 8.**
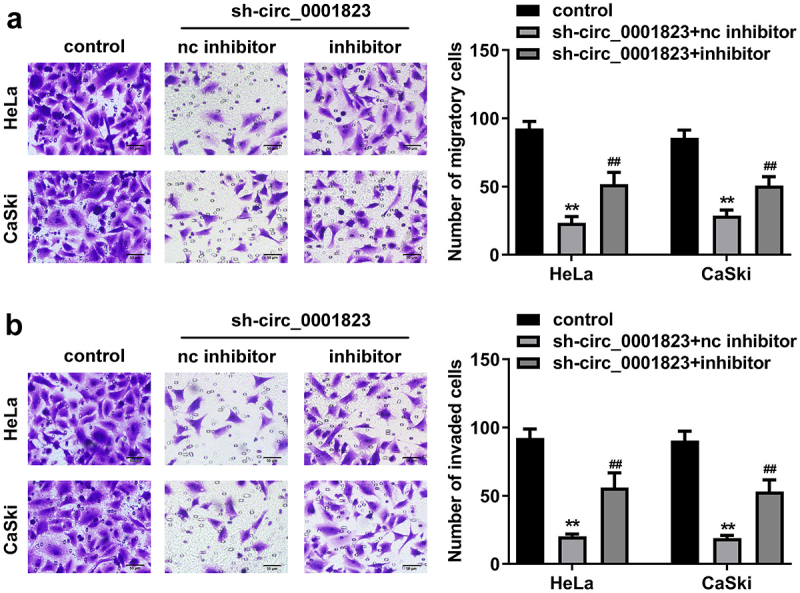
miR-613 inhibitor treatment inverted the effects of sh-circ_0001823 on the metastasis of the CC cells. A-B After sh-circ_0001823 as well as miR-613 inhibitor transfection, transwell assay was performed to detect the migration and invasion of the CC cells. **P < 0.01 VS control group. ##*P* < 0.01 VS sh-circ_0001823+ miR-613 inhibitor nc group.

The miR-613 expression was decreased after miR-613 inhibitor transfection and increased after miR-613 mimic transfection (Figure 7(a)). Furthermore, after sh-circ_0001823 and miR-613 inhibitor transfection, we found that miR-613 inhibitor reversed the effects of sh-circ_0001823 on the cell viability (Figure 7(b)), cloned cells number (Figure 7c), and the migrated (Figure 8(a)) and invaded cells (Figure 8b) in the CC cells.

## RAB8A is the target gene of miR-613

**Figure 9.**
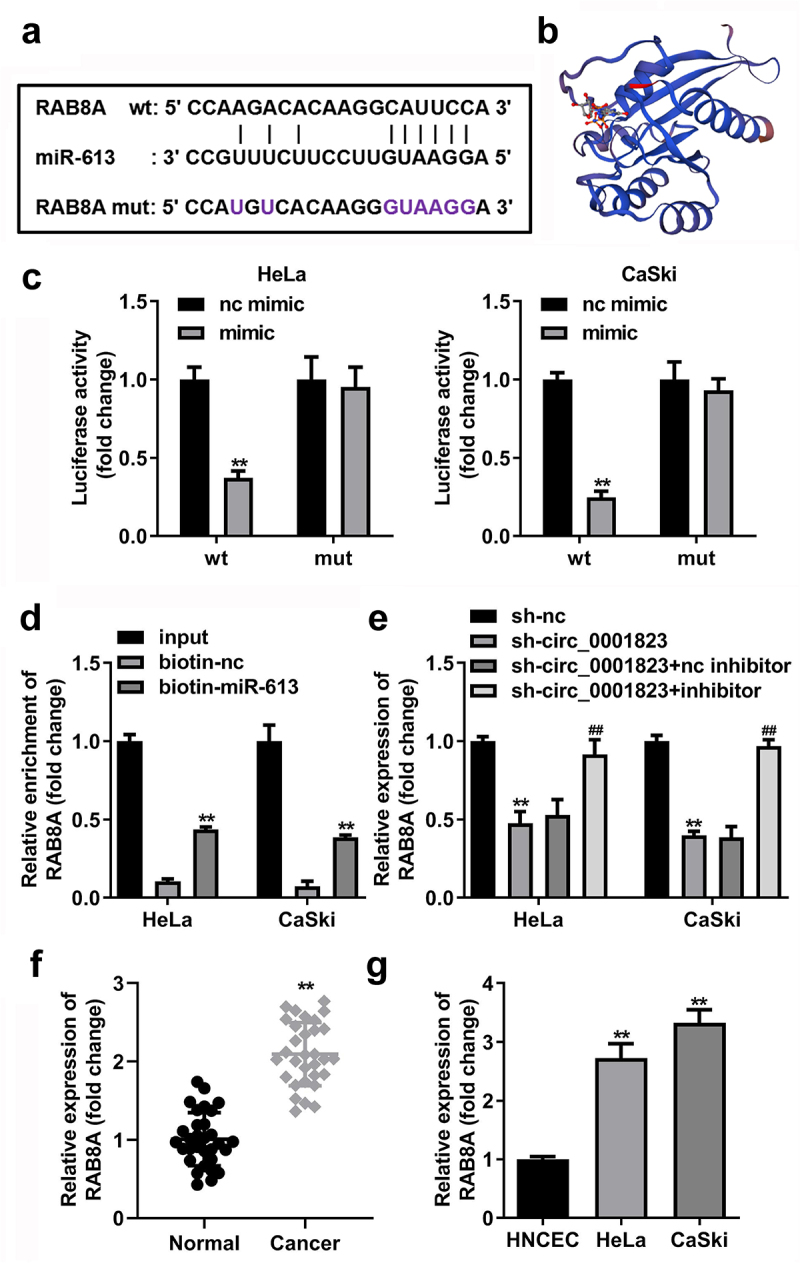
RAB8A targeted to miR-613 in CC cells. A The predicted RAB8A binding site in the miR-613 3’-UTR. B the 3D structure of RAB8A protein. C-D Double Luciferase Report and RNA pull-down assays were carried out to confirmed RAB8A could bind to miR-613. E The RAB8A expression in the CC cells was measured with qRT-PCR assay after sh-circ_0001823 and miR-613 inhibitor transfection. F-G The RAB8A expression in the CC tissues as well as cells was detected with qRT-PCR assay.

The binding site between RAB8A and miR-613 was obtained by online database TargetScan (Figure 9a), and the 3D structure of RAB8A protein is showed in Figure 9b. In addition, the luciferase activity was dramatically attenuated in the HeLa and CaSki cells incubated with RAB8A 3’ UTR wt (Figure 9c). In RNA-pull down assay, miR-613 enriched evidently higher level of RAB8A in the CC cells (Figure 9d). Besides, after sh-circ_0001823 transfection, the RAB8A expressions were dramatically decreased in the CC cells. And after miR-613 inhibitor transfection, the RAB8A expressions were dramatically increased (Figure 9e). Furthermore, we confirmed that RAB8A expressions were dramatically increased both in the CC tissues as well as cells (figure 9(f,g)).

RAB8A-silenced inhibited the malignant behavior of the CC cells

**Figure 10.**
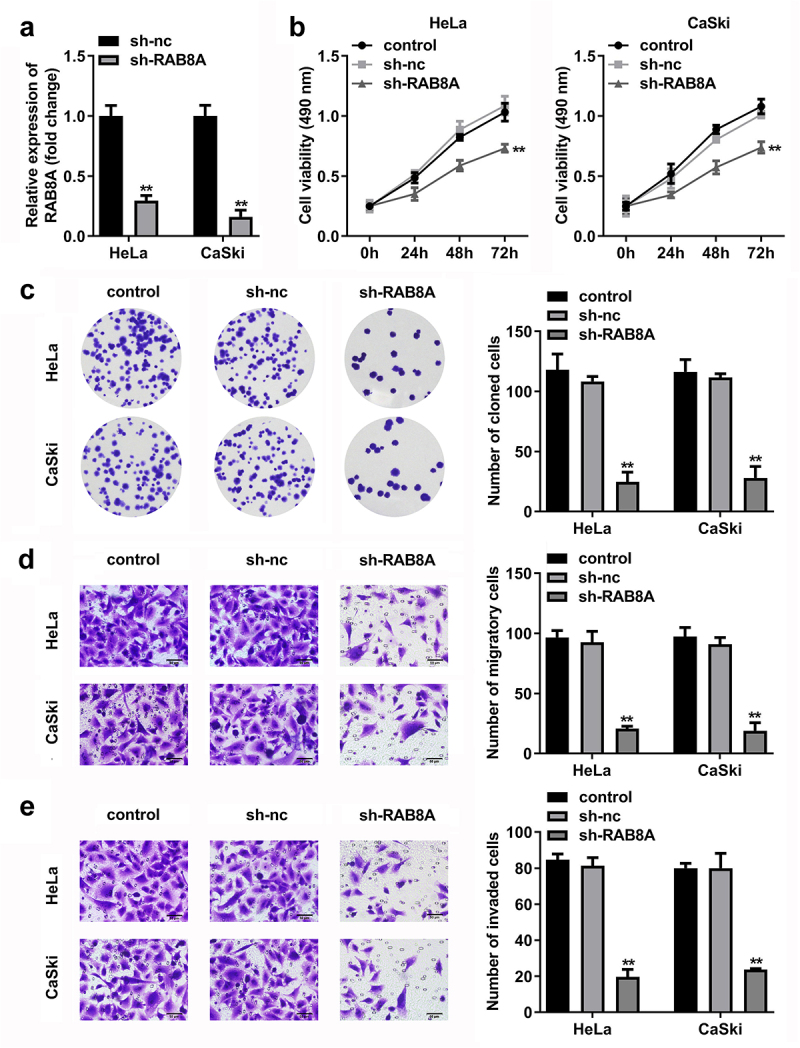
RAB8A-silenced inhibited the malignant behavior of the CC cells. A Validation of sh-RAB8A transfection efficiency. B-C After sh-RAB8A transfection, CCK-8 and colony formation assays were performed to measure the cell viability and cloned cells numbers. D-E After sh-RAB8A transfection, transwell assay was conducted to analyze the migration and invasion of the CC cells . **P < 0.01.

After sh-RAB8A transfection, the RAB8A expression was dramatically down-regulated in the HeLa and CaSki cells (Figure 10A). The cell viability and the number of cloned cells of the HeLa and CaSki cells were significantly down-regulated after sh-RAB8A transfection (Figure 10(B,c)). As shown in Figure 10(d,e), the migrated and invaded cells in the HeLa and CaSki cells were dramatically down-regulated after sh-RAB8A transfection.

## Over-expressed RAB8A inverted the role of miR-613 mimic in the CC cells

**Figure 11.**
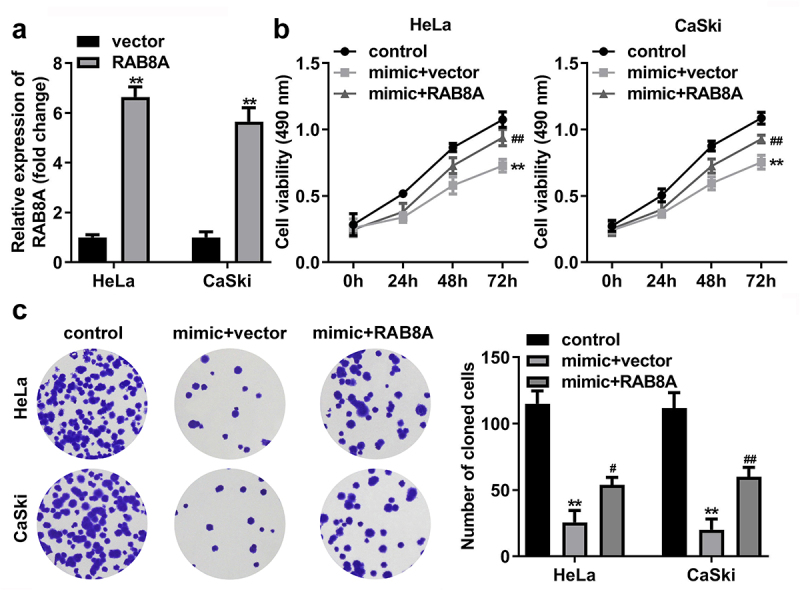
Over-expressed RAB8A inverted the effects of miR-613 mimic on the cell viability and cloned cells numbers of the CC cells. **A** Validation of RAB8A transfection efficiency. **B-C** After RAB8A and miR-613 mimic transfection, the cell viability and cloned cells numbers were measured by CCK-8 and colony formation assays. **P < 0.01 VS control group. #*P* < 0.05, ##*P* < 0.01 VS miR-613 mimic+vector group.

**Figure 12.**
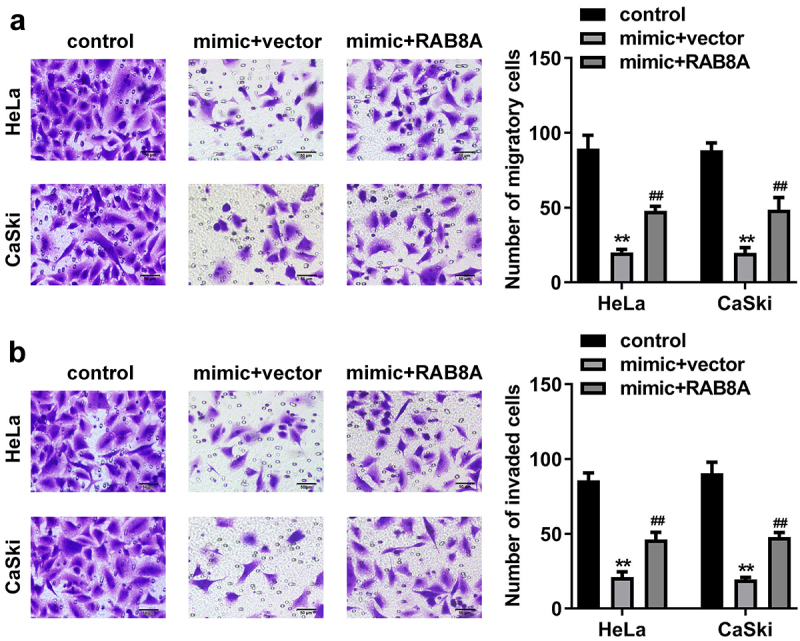
Over-expressed RAB8A inverted the effects of miR-613 mimic on the metastasis of the CC cells. **A-B** After RAB8A and miR-613 mimic transfection, the migration and invasion of the CC cells were determined with transwell assay. **P < 0.01 VS control group. ##*P* < 0.01 VS miR-613 mimic+vector group.

After RAB8A transfection, the RAB8A expression was significantly up-regulated (Figure 11A). In addition, miR-613 mimic treatment dramatically decreased the cell viability (Figure 11B), cloned cells number (Figure 11C), and the migrated (Figure 12A) and invaded cells (Figure 12B) in the CC cells. While overexpression of RAB8A antagonized the role of miR-613 mimic in the CC cells.

## Discussion

This study demonstrated that circ_0001823 was up-regulated in CC via bioinformatic analysis, which was further confirmed to be over-expressed in CC tissues as well as cells. Knockdown of circ_0001823 inhibited the growth and matastasis of the CC cells through modulating the miR-613/RAB8A axis.

Previously, circRNA is recognized as an aberrant RNA induced by wrong splicing of exon transcripts, which has not attracted people’s attention [24]. Until the 1990s, Nigro JM revealed that eukaryotic protein coding genes not only splice exon sequences together to form mature linear mRNA molecules, but also have a special back splicing reaction. This eventually leads to the reverse circularization of the downstream exon and the upstream exon and forms a single chain closed ring structure [25]. Recently, circRNAs were reported to be involved in the carcinogenesis and progression of various cancers, many researches confirmed that circRNAs could be promising diagnostic and prognostic biomarkers in cancers. For example, in breast cancer, circRNA_0025202 [26], circTADA2As [27], and circRNA_103809 [28] served as anti-oncogenics, while circSEPT9 [29], circ_001783 [30] and circDENND4C functioned as oncogenics. Similarly, in CC, Song et al. [31] demonstrated that abnormally expressed circRNA_101996 was bound up with poor prognosis of CC patients, and knockdown of circRNA_101996 restrained the malignant behavior of the CC cells. Additionally, circSLC26A4 [32], circ_0000515 [33] and circ_0000745 [34] were demonstrated to aggravate the progression of CC. Our research discovered a novel circRNA circ_0001823 and found that it was over-expressed in CC. Circ_0001823-silenced relieved the growth and metastasis of the CC cells. These results indicated that circ_0001823 might be potential therapeutic targets of CC.

A growing number of evidence has shown that circRNAs participate in CC progression as either oncogenes or tumor suppressors via many mechanisms, among which microRNA (miRNA) sponging is a common mechanism [31]. Further, circRNAs often acted as a competing endogenous RNA (ceRNA) to compete for miRNAs with mRNAs through miRNA response elements, which will regulate the expressions of target genes by miRNA and ultimately affect the biological behavior of tumor cells [35]. For instance, Luan et al. [36] confirmed that XLOC_006390 functioned as a ceRNA and negatively regulated the miR-331-3p and miR-338-3p expression of CC cells. In current research, through Double luciferase report and RNA pull-down assays, we confirmed that circ_0001823 served as a miR-613 sponge in CC cells. Mir-613 was originally reported to be involved in the regulation of lipid metabolism [37], and has been demonstrated to participate in the development of tumors now [38,39]. However, the mechanism of miR-613 in CC still needed to be further explored. Current study demonstrated miR-613 was down-regulated in the CC tissues as well as cells. We confirmed that knockdown of miR-613 reversed the effects of sh-circ_0001823 on the malignant behaviors of the CC cells. These findings were similar to those of previous studies, which confirmed miR-613 was reversely regulated by circRNAS [40,41].

Finally, via bioinformatic analysis, RAB8A was predicated and confirmed to be target mRNA of miR-613. RAB8A is a kind of multifunctional GTPase, which combines with a variety of effectors and plays a role in different cellular pathways [42]. Furthermore, RAB8A can affect the transport of vesicles between the Trans Golgi network and the plasma membrane, and participate in the regulation of the anchoring of glucose transporter 4 [43]. Zhang et al. [44] reported that miR-30d-5p regulated the trophoblast cell functions via targeting RAB8A expression, which may provide a novel vision for the treatment of gestational diabetes mellitus. However, there are few reports on the role of RAB8A in cancer progression. Previous study confirmed RAB8A was up-regulated in endometrial carcinoma, which may be an effective tumor marker [45]. In current research, we demonstrated that RAB8A was over-expressed in the CC tissues as well as cells. Furthermore, the binding relationship between miR-613 and RAB8A was confirmed with double luciferase report and RNA pull-down assays. Over-expressed RAB8A inverted the role of miR-613 mimics in the CC cells. All these results implied that circ_0001823 aggravated the CC development through modulating the miR-613/RAB8A axis. However, there are still some limitations in this study. Due to limited conditions, only 30 patients were screened for this study. We will collect more samples in our future research.

## Conclusion

To sum up, this study demonstrated that circ_0001823 and RAB8A were over-expressed, and miR-613 was down-regulated in the CC cells and tissues. Knockdown of circ_0001823 inhibited the malignant behavior of the CC cells via regulating the miR-613/RAB8A axis.
